# Dual-functional sulfonated PEEK implants via graphene oxide–mediated BMP-2 gene delivery: enhanced osteogenic and antibacterial performance *in vitro*

**DOI:** 10.3389/fmed.2026.1763692

**Published:** 2026-03-04

**Authors:** Yueying Pan, Bumairemu Yiminjiang, Adilai Abula, Refeina Tuerxun, Maihefuzi Aishan

**Affiliations:** 1 Department of Prosthodontics and Dental Implantology School, Hospital of Stomatology, The First Affiliated Hospital of Xinjiang Medical University, Urumqi, China; 2 Department of Oral Surgery School, Hospital of Stomatology, The First Affiliated Hospital of Xinjiang Medical University, Urumqi, China; 3 Stomatological Research Institute of Xinjiang Uygur Autonomous Region, Urumqi, China

**Keywords:** antibacterial, bonemorphogenetic protein-2, gene delivery, graphene oxide, osteogenesis, polyetheretherketone

## Abstract

**Introduction:**

Polyetheretherketone (PEEK) is a promising orthopedic implant material due to its bone-mimetic mechanical properties; however, its bioinert surface and susceptibility to bacterial colonization limit its clinical efficacy. Strategies to enhance osteointegration while providing antibacterial functionality are urgently needed.

**Methods:**

Graphene oxide (GO) was functionalized with polyethylene glycol (PEG) and complexed with a plasmid encoding bone morphogenetic protein-2 (pBMP-2) to form a GO-PEG/pBMP-2 complex. This complex was coated onto sulfonated PEEK (SPEEK) via freeze-drying, yielding SPEEK-(GO-PEG/pBMP-2) composites. The materials were systematically compared with pristine PEEK, SPEEK, and SPEEK-GO-PEG controls. Surface chemistry, morphology, hydrophilicity, gene loading efficiency, and coating uniformity were characterized. In vitro assessments included cell adhesion, proliferation, viability, alkaline phosphatase (ALP) activity, mineralized matrix deposition, osteogenic gene expression, and antibacterial activity against *Staphylococcus aureus* and *Escherichia coli*.

**Results:**

Successful PEGylation of GO and efficient pBMP-2 loading were confirmed, with uniform coating and significantly improved hydrophilicity on SPEEK-(GO-PEG/pBMP-2). This composite markedly enhanced osteoblast adhesion, proliferation, and viability, along with elevated ALP activity, increased mineralized nodule formation, and upregulated expression of key osteogenic genes (e.g., Runx2, OPN, OCN). Both GO-containing coatings—SPEEK-GO-PEG and SPEEK-(GO-PEG/pBMP-2)—exhibited strong antibacterial effects against both Gram-positive and Gram-negative bacteria, with no significant difference attributable to BMP-2 loading.

**Conclusion:**

The SPEEK-(GO-PEG/pBMP-2) platform simultaneously promotes osteogenesis through localized BMP-2 gene delivery and provides robust antibacterial protection via GO. This dual-functional design addresses two major limitations of PEEK implants, offering a promising strategy for next-generation orthopedic biomaterials.

## 1 Introduction

Polyetheretherketone (PEEK), a semi-crystalline thermoplastic within the polyaryletherketone family, has emerged as a promising alternative for bone implants (1). Its mechanical properties closely match those of human cortical bone, reducing stress shielding, while its radiolucency allows for clear postoperative imaging (2). PEEK also boasts superior high-temperature stability, corrosion resistance, and mechanical durability, making it a highly attractive material in orthopedic and dental implantology (3).

Despite these favorable physicochemical and mechanical properties, achieving consistent clinical success with PEEK-based implants remains a significant and multifactorial challenge. Among the various limitations reported, insufficient osseointegration at the bone–implant interface and implant-associated infections are widely recognized as the primary barriers to long-term implant performance (4).

The mechanisms underlying the limited osseointegration of PEEK have been extensively investigated. Previous studies have demonstrated delayed or inadequate bone–implant integration when PEEK is employed as a load-bearing implant material, particularly under osteoporotic conditions or in compromised bone microenvironments (5). These unfavorable outcomes are primarily attributed to the intrinsic hydrophobicity and biological inertness of PEEK, both of which impede effective osseointegration (6). Such surface characteristics restrict early protein adsorption and cell adhesion, subsequently impairing new bone deposition, stable interfacial anchorage, and increasing the risk of implant micromotion and long-term loosening (7).

Concurrently, implant-associated infections are closely linked to bacterial adhesion, aggregation, and biofilm formation on implant surfaces, processes that markedly reduce antibiotic efficacy and challenge host immune clearance (8). Once established, these infections can result in severe clinical complications, including delayed union or nonunion of fractures, as well as implant loosening or failure (9). Due to its biologically inert surface, unmodified PEEK is particularly susceptible to bacterial colonization and biofilm development, which may trigger persistent local inflammatory responses and ultimately compromise implant stability and long-term clinical outcomes (10).

Consequently, the simultaneous enhancement of osteogenic activity and antimicrobial performance through effective surface modification strategies has become a critical objective for improving the clinical applicability of PEEK in bone regeneration and implant-related applications.

Graphene oxide (GO), a two-dimensional honeycomb-structured nanomaterial composed of sp^2^-hybridized carbon atoms, exhibits exceptional chemical, optical, electrical, and mechanical properties (11). Its basal planes and edges are enriched with functional groups such as epoxy, hydroxyl, and carboxyl, which serve as versatile anchoring sites for biomolecules including proteins and nucleic acids (12). These functional moieties endow GO with remarkable chemical and biological functionalization capacity, enabling efficient loading and targeted delivery of therapeutic agents. Functionalized GO derivatives have been extensively investigated as nucleic acid carriers, where they not only facilitate DNA/RNA loading and transfection but also protect nucleic acids from enzymatic degradation, thereby markedly improving transfection efficiency (13). Moreover, GO demonstrates broad-spectrum antimicrobial activity against both Gram-negative and Gram-positive bacteria (14), underscoring its potential as a key material in antimicrobial therapies.

To improve the biocompatibility of graphene oxide (GO), researchers have functionalized its surface with hydrophilic and biocompatible biomolecules. Among these strategies, polyethylene glycol (PEG) modification, achieved through covalent bonding, has shown outstanding gene transfection efficiency and excellent biocompatibility (15). Consequently, PEG-functionalized GO is regarded as a highly promising platform for biomolecular delivery, particularly for DNA and RNA therapeutics (16–18).

Bone morphogenetic protein-2 (BMP-2) is one of the most extensively studied osteoinductive growth factors, playing a crucial role in bone regeneration by promoting osteoblast differentiation and enhancing bone matrix formation (19). However, clinical use of recombinant BMP-2 is often limited by its rapid degradation, short half-life, and potential side effects, such as ectopic bone formation (20). Gene delivery systems offer a promising alternative by enabling localized expression of BMP-2 in target tissues (21). In this context, the combination of GO-PEG as a gene carrier with pBMP-2 provides an effective strategy for achieving osteoinductive stimulation while potentially reducing systemic side effects.

In this study, we harnessed the DNA delivery capability of PEG-functionalized GO by grafting the BMP-2 gene onto its surface and subsequently integrating it with sulfonated PEEK. *In vitro* cellular assays confirmed that BMP-2 gene transfection stimulated host cells to upregulate osteogenesis-related genes, thereby promoting the interfacial integration between the implant material and surrounding bone tissue. This strategy not only accelerated the osseointegration of PEEK at the implantation site but also markedly enhanced the overall quality of bone–implant integration.

In addition, *in vitro* antimicrobial assays demonstrated that the antibacterial activity of GO on the PEEK surface effectively suppressed the growth and proliferation of *Staphylococcus aureus* (*S. aureus*) and *Escherichia coli* (*E. coli*) both on the implant surface and in the surrounding environment. This modification endowed PEEK with significant antimicrobial capability *in vitro*. Through this dual-functional design, the present study not only enhanced the osseointegration capacity of PEEK but also reinforced its antibacterial properties, providing new insights into the development of next-generation bone implant materials.

## 2 Materials and methods

### 2.1 Preparation and characterization of GO-PEG

GO powder (Cat. No. NM000060, Solarbio, Beijing, China) was dispersed in deionized water via ultrasonication to obtain a homogeneous suspension. The suspension was subjected to carboxylation at 4 °C, followed by repeated centrifugation and washing until the pH stabilized at 7.0–7.5. Subsequently, the GO suspension was mixed with pre-dissolved 6-NH₂-PEG solution and further ultrasonicated. To promote covalent conjugation, 1.0 mg/mL of 1-ethyl-3-(3-dimethylaminopropyl) carbodiimide (EDC) was added, and the reaction mixture was stirred at room temperature to obtain the GO-PEG composite solution.

Functional groups were analyzed by Fourier transform infrared spectroscopy (FTIR; Thermo Electron Corporation, United States) over a spectral range of 4,000–400 cm^−1^. Microstructural morphology was examined using transmission electron microscopy (TEM; Thermo Electron Corporation, United States) at 16,500 × magnification. Elemental composition was determined using an organic elemental analyzer (vario MICRO cube, Elementar, Germany) in CHN mode. Thermogravimetric analysis (TGA; STA 449G, Germany) was performed from room temperature to 500 °C at a heating rate of 15 °C/min.

### 2.2 Preparation and characterization of SPEEK-(GO-PEG/pBMP-2)

The pBMP-2 plasmid (Cat. No. P40382, MiaoLingBio, Wuhan, China) was dissolved in sterile water according to the manufacturer’s instructions and mixed with GO-PEG solution at nitrogen-to-phosphate (N/P) ratios of 10, 20, and 40. The mixtures were incubated at room temperature to form GO-PEG/pBMP-2 complexes.

Plasmid loading efficiency was determined by measuring unbound pBMP-2 in the supernatant after washing. The concentration of free plasmid was quantified using a dsDNA fluorescence assay kit (Cat. No. SY0259, Bio-Lab, Beijing, China) following the manufacturer’s protocol. Fluorescence intensity was measured using a fluorescence spectrophotometer (RF-1501, Shimadzu, Japan), and concentrations were calculated from a standard curve.


$$\begin{array}{l} \text{Plasmid loading}\;(\%)=\\ {}[\begin{array}{l} (\text{Total pBMP}-2\;\text{input}-\text{Free pBMP}-2)/\\ \text{Total complex mass} \end{array}]\times 100\% \end{array}$$

PEEK disks (Victrex plc, Thornton Cleveleys, United Kingdom) were immersed in 98% sulfuric acid at room temperature and sulfonated under magnetic stirring (100 rpm) for 5 min (22). Samples were immediately removed and ultrasonically cleaned alternately in deionized water and acetone to remove residual acid (23). After sterilization and drying, sulfonated PEEK (SPEEK) was obtained.

For *in vitro* release study, SPEEK-(GO-PEG/pBMP-2) samples were immersed in 1 mL of PBS (pH 7.4) at 37 °C with shaking (100 rpm). At predetermined time points (1, 3, 5, 7, 10, 14, 21, 28, and 35 days), the entire medium was collected and replaced with fresh PBS. Samples were stored at −20 °C until analysis. The amount of released pBMP-2 was quantified using the same dsDNA quantitation kit described above, with concentrations calculated from a standard curve prepared using known amounts of pBMP-2.

Surface morphology was observed using field emission scanning electron microscopy (SEM, JSM-7500F, JEOL, Japan). Surface hydrophilicity was assessed by measuring the static water contact angle (CA, JY-82B, Kruss DSA, Germany) under ambient temperature and humidity.

### 2.3 Isolation and identification of rat bone marrow–derived mesenchymal stem cells

A total of 20 male Sprague–Dawley (SD) rats (6-week-old, 135–150 g), provided by the Animal Research Center of Xinjiang Medical University, were used for the isolation of bone marrow–derived mesenchymal stem cells. rBMSCs were isolated from each individual rat and cultured independently without pooling, thereby preserving distinct biological origins. All SD rats were fed with redistilled water and sterilized food and housed in a pathogen-freeroom with environmental conditions of good ventilation, 55 + 5%humidity, 20 + 2 °C temperature, and proper light intensity (4 W/m^2^) for 12 h per day. Six weeks later, all rats were killed using a rat euthanasia box filled with carbon dioxide (LC-800-S1, Shanghai Yuyan Scientific Instrument, China), and their femurs and tibias were harvested together with surrounding soft tissues. Both ends of each bone were removed to expose the medullary cavity, and bone marrow was aspirated using complete culture medium. The marrow was repeatedly flushed to ensure maximal cell recovery. The collected suspension was centrifuged at high speed, the supernatant discarded, and the pellet resuspended in fresh medium. Cells were cultured in a humidified incubator at constant temperature, with medium replaced on the third day after primary isolation. Experiments were performed using cells at the third passage. All sections of this protocol are conformed to the Animal Research Reporting *in Vivo* Experiments (ARRlVE) guidelines and approved by the Ethics Committee at the First Affiliated Hospital of Xinjiang Medical University (approval no. IACUC-JT-20230515-09).

### 2.4 Biocompatibility assessment of SPEEK-(GO-PEG/pBMP-2)

Rat BMSCs were seeded onto different material samples in 24-well plates. After 3 days of culture, cells were fixed with 3% glutaraldehyde for 3 h, dehydrated through a graded ethanol series, and dried. Cell adhesion and morphology on the material surfaces were examined by field emission scanning electron microscopy (FE-SEM, JSM-7500F, JEOL, Japan).

The cytoskeleton of rBMSCs was visualized using confocal laser scanning microscopy (CLSM, Nikon C2, Japan). Cells were permeabilized with 0.1% Triton X-100 (Cat. No. IR9073, Solarbio, Beijing, China) for 5 min, followed by staining with rhodamine–phalloidin (Cat. No. CA1620, Solarbio, Beijing, China) for F-actin and 4′,6-diamidino-2-phenylindole (DAPI; Cat. No. C0065, Solarbio, Beijing, China) for nuclei.

Cell viability was evaluated using the Cell Counting Kit-8 (CCK-8Cat. No. C0038, Beyotime, Shanghai, China). At days 1, 3, 5, and 7 of culture, a mixture of serum-free medium and CCK-8 reagent (10:1) was added to each well of 96-well plates. After incubation, the optical density (OD) was measured at 450 nm using a microplate reader.

Cytotoxicity was further assessed by Live/Dead staining. After 3 days of culture, 250 μL of a Calcein AM/PI working solution (Cat. No. C1371M, Beyotime, Shanghai, China) was added to the cells and incubated at 37 °C in the dark for 30 min. Stained cells were then visualized using a laser confocal microscope.

### 2.5 Osteogenic potential of SPEEK-(GO-PEG/pBMP-2)

The osteogenic differentiation of rBMSCs on different materials was evaluated by alkaline phosphatase (ALP) staining and activity assays. Cells were cultured in osteogenic induction medium (Cat. No. PD-008, Procell, Wuhan, China) for 7 and 14 days, respectively. After culture, cells were fixed with 4% paraformaldehyde and stained with a BCIP/NBT staining solution (Cat. No. C3206, Beyotime, Shanghai, China). Following incubation in the dark, stained samples were imaged and analyzed using a stereomicroscope.

Cells were lysed by adding 1 mL of 1% Triton X-100 solution to each well and gently pipetting. After centrifugation, the supernatant was collected, and ALP activity in the lysates was determined using an ALP activity assay kit (Cat. No. P0321S, Beyotime, Shanghai, China) according to the manufacturer’s instructions. Absorbance was measured at 405 nm with a microplate reader.

The osteogenic potential of the materials was further evaluated by mineralized nodule staining and quantitative analysis. Cells were cultured in osteogenic induction medium for 14 and 21 days, respectively. After culture, cells were fixed with 4% paraformaldehyde and stained with an Alizarin Red S solution (Cat. No. C0148S, Beyotime, Shanghai, China) for 30 min, followed by thorough rinsing. Extracellular matrix mineralization nodules were imaged using a stereomicroscope. For quantification, a 10% cetylpyridinium chloride solution (Cat. No. Y175536, Beyotime, Shanghai, China) was added to dissolve the bound dye. After incubation for 1 h and gentle mixing, appropriate volumes of the eluates were transferred to a 96-well plate, and absorbance was measured at 562 nm with a microplate reader.

The expression of osteogenesis-related genes, including runt-related transcription factor 2 (Runx2), ALP, type I collagen (COL-1), and osteopontin (OPN), was analyzed by quantitative real-time PCR (qRT-PCR), with GAPDH serving as the housekeeping gene. After 7 days of culture in osteogenic induction medium, total RNA was extracted from each group using Trizol reagent and reverse-transcribed into cDNA. PCR amplification was conducted under the following conditions: pre-denaturation at 95 °C for 3 min; denaturation at 95 °C for 10 s; annealing/extension at 60 °C for 25 s; 40 cycles in total. Gene expression levels were quantified using a qRT-PCR kit with gene-specific primers (sequences listed separately). Relative expression was calculated by the 2^(-ΔΔCt) method, and statistical analysis was performed. All experiments were performed with three independent biological replicates (*n* = 3) (Table 1).

**Table 1:** Sequences of the forward and reverse primers of the tested genes.

Genes	Sequence (5′–3′)
BMP-2	F: 5′-GCCAAACACAAACAGCGGAAGC-3′
	R: 5′-GGCCACGATCCAGTCATTCCAC-3′
Col-1	F: 5′-TGTTGGTCCTGCTGGCAAGAATG-3′
	R: 5′-GTCACCTTGTTCGCCTGTCTCAC-3′
Alp	F: 5′-GTGCGGTACTGACTGGGAATGC -3′
	R: 5′- CAGGCTTCTTCTTCACTGGTCCAC-3′
Runx2	F: 5′-TCCGCCACCACTCACTACCAC-3′
	R: 5′- GGAACTGATAGGACGCTGACGAAG-3′
OPN	F: 5′- GACGATGATGACGACGACGATGAC-3′
	R: 5′- GTGTGCTGGCAGTGAAGGACTC-3′
GAPDH	F: 5′-CAGGGCTGCCTTCTCTTGTG-3
	R: 5′-GATGGTGATGGGTTTCCCGT-3′

### 2.6 Antimicrobial activity of SPEEK-(GO-PEG/pBMP-2)

The antibacterial activity of the materials was evaluated using a dilution plating method. Four groups of samples—PEEK, SPEEK, SPEEK-GO-PEG, and SPEEK-(GO-PEG/pBMP-2)—were placed in 12-well plates. Bacterial suspensions of *Escherichia coli* and *Staphylococcus aureus* were adjusted to an OD₆₀₀ of 0.15, and 1 mL of each suspension was added to the wells for co-culture at 37 °C for 24 h. After incubation, the suspensions were removed, diluted to 10^6^ CFU/mL in LB broth, and 30 μL of each dilution was spread evenly onto LB agar plates using sterile glass beads. Plates were incubated at 37 °C for 24 h, and the number of colonies was counted.

Simultaneously, bacterial adhesion and morphology on the material surfaces were examined by scanning electron microscopy (SEM). Samples from each group were incubated with 1 mL of *E. coli* or *S. aureus* suspension at 37 °C for 24 h to allow biofilm formation. After incubation, samples were washed with PBS, fixed, dehydrated as described previously, dried at room temperature, and sputter-coated with gold. Bacterial morphology on the sample surfaces was observed under SEM at a magnification of 5,000 × .

### 2.7 Statistical analysis

Statistical analyses were performed using SPSS 26.0 and GraphPad Prism 9. All experiments were performed with three independent biological replicates (*n* = 3). Data are expressed as mean ± standard deviation (SD) for continuous variables with normal distribution and homogeneity of variance. Comparisons between two groups were conducted using independent-samples t tests, while comparisons among multiple groups were assessed by one-way analysis of variance (ANOVA) followed by least significant difference (LSD-t) *post-hoc* tests. A *p* < 0.05 was considered statistically significant.

## 3 Results

### 3.1 Characterization of GO-PEG

**Figure 1:**
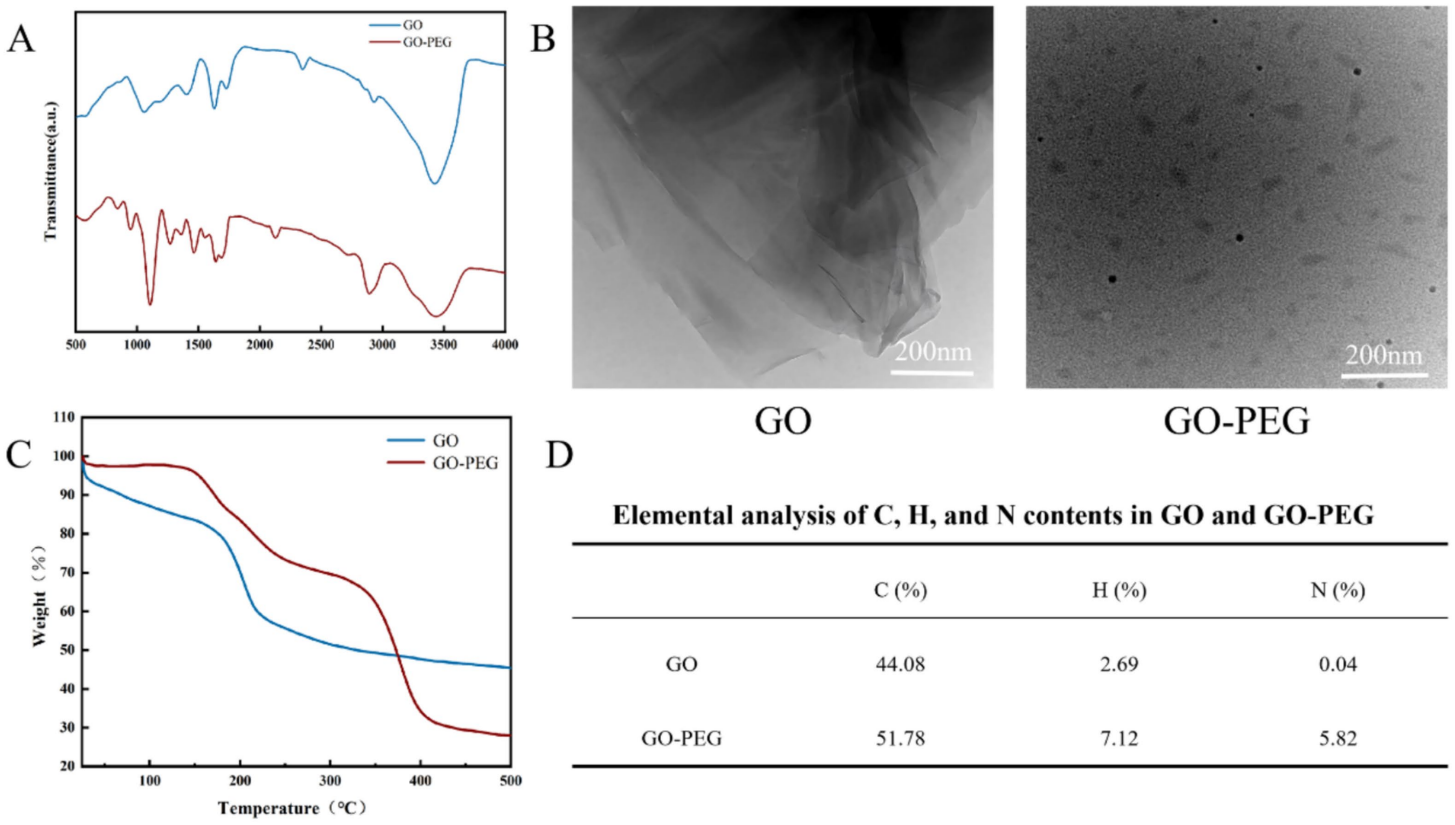
Characterization of GO and GO-PEG. **(A)** FTIR spectra of GO and GO-PEG. **(B)** TEM images of GO and GO-PEG. **(C)** Thermogravimetric analysis (TGA) curves of GO and GO-PEG. **(D)** Elemental analysis of carbon (C), hydrogen (H), and nitrogen (N) in GO and GO-PEG.

FTIR confirmed the functionalization of GO with PEG. In the infrared spectrum, characteristic absorption peaks were observed at 2,800–2,900 cm^−1^ (C–H stretching), 1,736 cm^−1^ (C=O stretching), and 3,427 cm^−1^ (O–H stretching). Compared with GO, GO-PEG exhibited marked spectral changes, most notably a pronounced increase in the C–H absorption band at ~2,800 cm^−1^, indicating successful PEG modification of the GO surface (Figure 1A).

TEM revealed distinct morphological differences between GO and GO-PEG. GO exhibited an irregular polygonal shape with a multilayered, wrinkled structure. Following PEG modification, the nanosheets displayed a more circular morphology and a markedly reduced particle size, confirming substantial structural changes (Figure 1B).

The PEG loading on the GO surface was quantified by combining TGA with EA. TGA revealed the temperature-dependent mass loss profiles of GO and GO-PEG (Figure 1C). EA showed the elemental compositions of C, H, and N for both groups (Figure 1D). Notably, the nitrogen content increased from 0.04% in GO to 5.82% in GO-PEG, corresponding to a PEG loading of approximately 16.8%.

### 3.2 Transfection efficiency and biocompatibility of GO-PEG/pBMP-2

**Figure 2:**
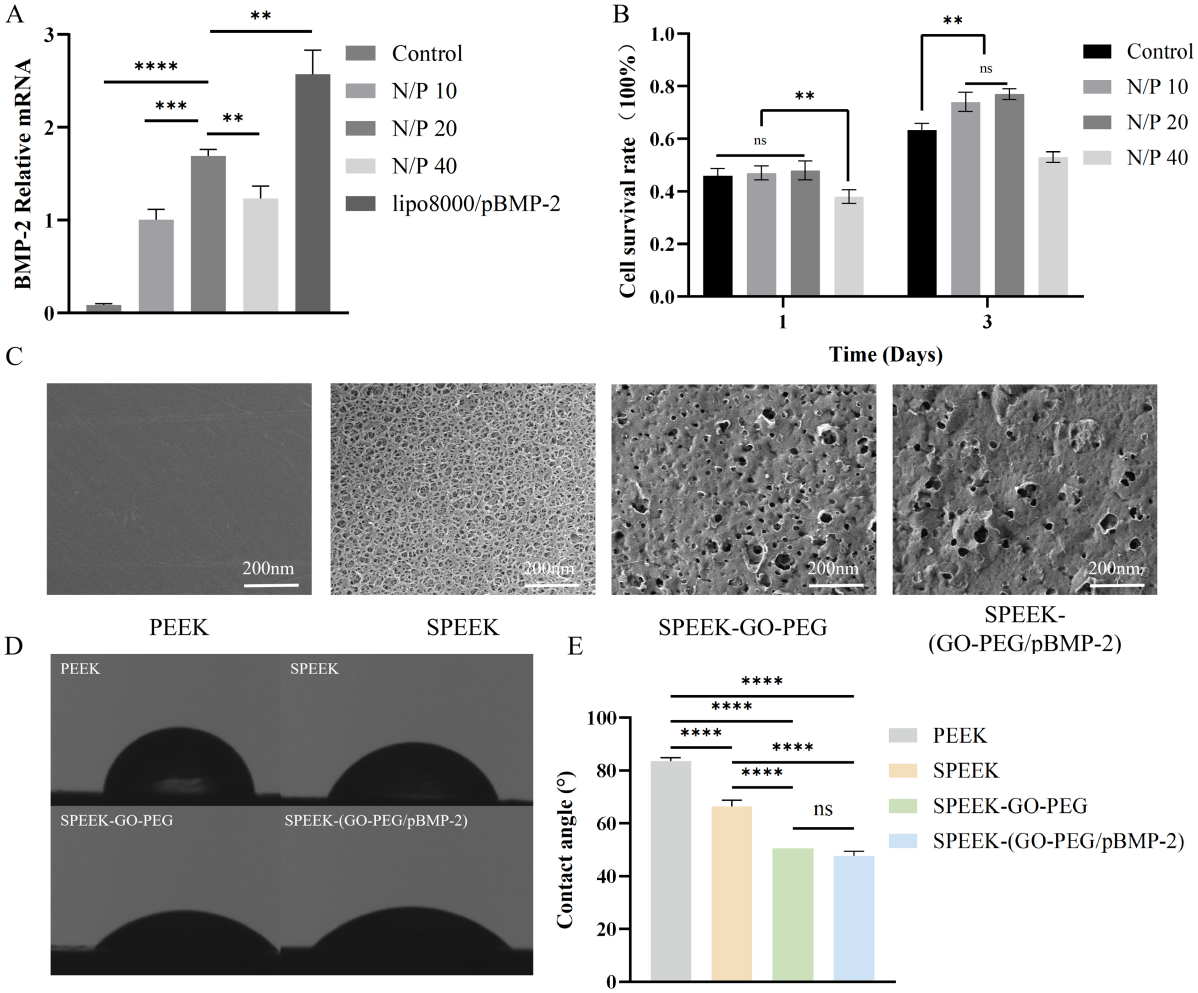
Transfection, cytocompatibility of GO-PEG/pBMP-2, and characterization of SPEEK-(GO-PEG/pBMP-2). **(A)** Transfection efficiency at different N/P ratios measured by qRT-PCR. **(B)** Cell viability at different N/P ratios evaluated by CCK-8 assay. **(C)** Representative SEM images of material surfaces. **(D)** Static water contact angle measurements of each group. **(E)** Quantitative analysis of contact angles (ns, *p* > 0.05; **p < 0.01; ***p < 0.001; ****p < 0.0001).

The transfection efficiency of pBMP-2 at different N/P ratios (10, 20, 40) was evaluated by qRT-PCR. pBMP-2 effectively transfected cells and enhanced BMP-2 mRNA expression across all N/P ratios tested. Expression levels increased progressively from N/P 10 to N/P 20, indicating improved transfection efficiency (*p* < 0.001). Compared with the positive control (lipo8000/pBMP-2), the N/P 20 group showed slightly lower efficiency (*p* < 0.01). Nevertheless, BMP-2 mRNA expression in all pBMP-2 groups was significantly higher than in the untreated control (*p* < 0.0001) (Figure 2A).

The cytocompatibility of GO-PEG/pBMP-2 transfection complexes at different N/P ratios was assessed by CCK-8 assay. On Day 1, no significant differences in cell viability were observed between the N/P 10 and N/P 20 groups compared with the control, and no statistical difference was detected between these two groups. In contrast, viability in the N/P 40 group was significantly reduced (*p* < 0.01). By Day 3, cell viability in the N/P 10 and N/P 20 groups increased relative to the control (*p* < 0.01), with no significant difference between them, whereas the N/P 40 group remained markedly reduced (Figure 2B). These results suggest that N/P ratios of 10–20 provide favorable biocompatibility, while higher ratios substantially impair cell viability. Accordingly, the N/P 20 group was selected for subsequent experiments.

### 3.3 Characterization of SPEEK-(GO-PEG/pBMP-2)

In this study, the plasmid loading efficiency of the SPEEK-(GO-PEG/pBMP-2) composite was 1.4% ± 0.5%. The *in vitro* release profile of pBMP-2 showed an initial burst release, followed by a sustained and controlled release. The cumulative release reached approximately 79% by day 30, indicating that the SPEEK-(GO-PEG/pBMP-2) composite successfully achieved long-term sustained release of pBMP-2 from a PEEK-based implant system (Supplementary Figure S1).

SEM was used to examine the surface morphology of samples in each group. PEEK exhibited a relatively flat and smooth surface with only minor polishing scratches, whereas sulfonated SPEEK displayed a distinct three-dimensional porous structure. After coating with GO-PEG or GO-PEG/pBMP-2, the porous structure was partially covered, and layered functionalized graphene oxide was observed on both surfaces, with no marked differences between the two groups (Figure 2C).

The surface hydrophilicity of each group was evaluated by water CA measurements. Untreated PEEK exhibited the largest contact angle, indicating strong hydrophobicity. In contrast, sulfonated PEEK showed a markedly reduced contact angle (*p* < 0.0001), reflecting enhanced hydrophilicity. Further coating with GO-PEG or GO-PEG/pBMP-2 decreased the contact angle even more (*p* < 0.0001), demonstrating superior hydrophilicity, although no significant difference was detected between the two groups (Figures 2D,E).

### 3.4 Biocompatibility of SPEEK-(GO-PEG/pBMP-2)

**Figure 3:**
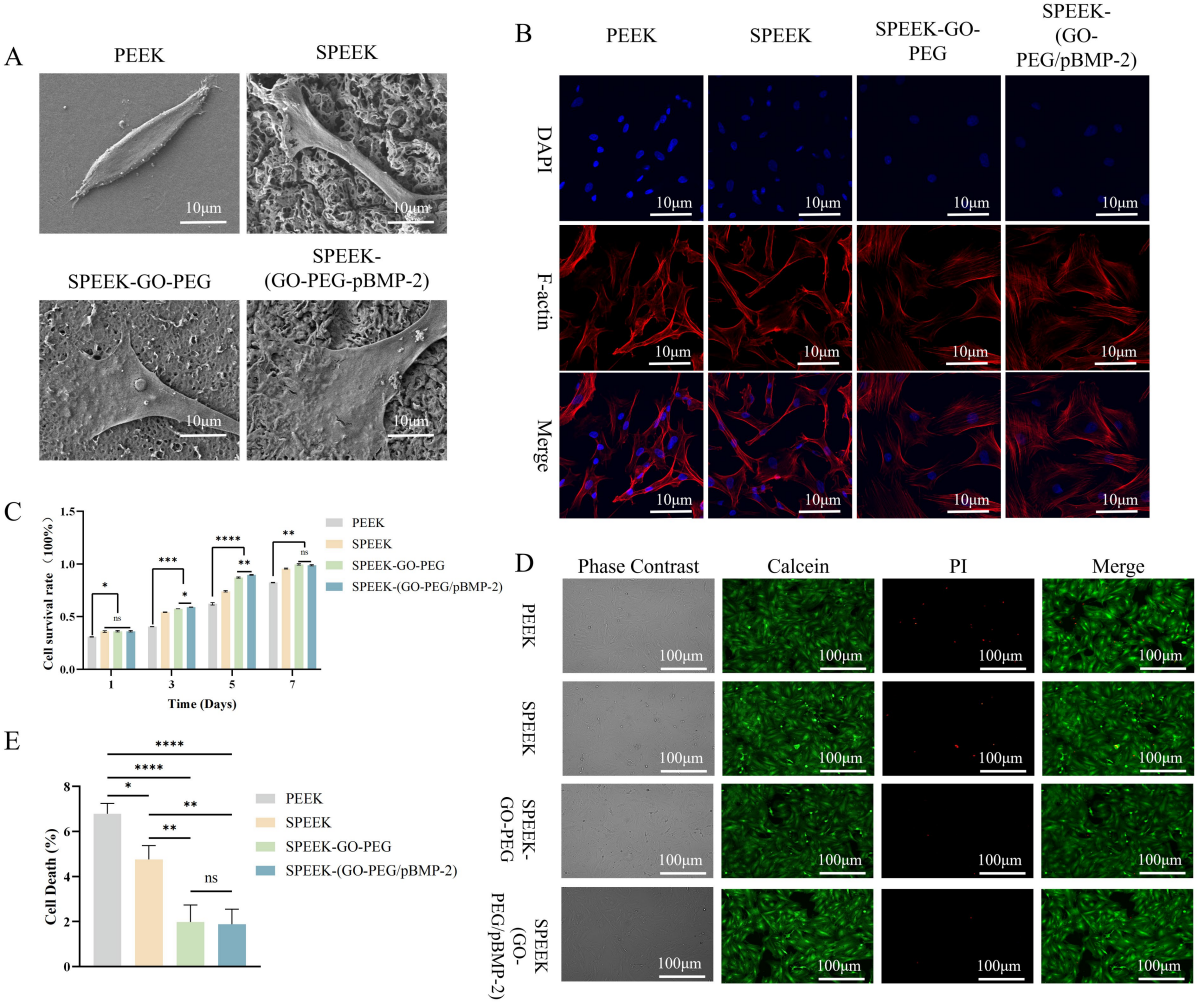
Biocompatibility of SPEEK-(GO-PEG/pBMP-2). **(A)** Representative SEM images of rBMSC adhesion and morphology. **(B)** Cytoskeletal organization visualized by rhodamine–phalloidin (F-actin, red) and DAPI (nuclei, blue) staining. **(C)** Cell proliferation evaluated by CCK-8 assay. **(D)** Live/Dead staining illustrating cytotoxicity. **(E)** Quantitative analysis of Live/Dead staining (ns, *p* > 0.05; *p < 0.05; **p < 0.01; ***p < 0.001; ****p < 0.0001).

SEM observation of rBMSCs cultured on different material surfaces revealed distinct morphological differences. On PEEK, cells exhibited a slightly elongated spindle shape with few peripheral filopodia. In contrast, cells on SPEEK displayed pronounced spreading, with filopodia extending from the cell edges and anchoring to the porous surface. On SPEEK-GO-PEG and SPEEK-(GO-PEG/pBMP-2), cells demonstrated extensive spreading with lamellipodia formation, larger cell areas, and strong adhesion to the material surfaces (Figure 3A).

Fluorescent staining of rBMSCs was performed with Rhodamine–Phalloidin (red) for F-actin and DAPI (blue) for nuclei to evaluate cell adhesion on different material surfaces. On PEEK and SPEEK, cells predominantly displayed a spindle-like morphology with visible F-actin filaments arranged in a relatively disordered pattern. In contrast, cells on SPEEK-GO-PEG and SPEEK-(GO-PEG/pBMP-2) exhibited well-spread, polygonal morphologies. F-actin expression was markedly increased and organized into a regular network aligned with the direction of cell extension (Figure 3B).

Cell proliferation on different materials was evaluated by CCK-8 assay at days 1, 3, 5, and 7. At all-time points, proliferation in the SPEEK, SPEEK-GO-PEG, and SPEEK-(GO-PEG/pBMP-2) groups was significantly higher than that in the PEEK control (*p* < 0.05). No significant differences were observed among the three modified groups on day 1. From day 3 onward, proliferation in the SPEEK-GO-PEG and SPEEK-(GO-PEG/pBMP-2) groups was markedly greater than in the SPEEK group. Moreover, at days 3 and 5, cell numbers in the SPEEK-(GO-PEG/pBMP-2) group exceeded those in the SPEEK-GO-PEG group (*p* < 0.01), indicating superior cell viability (Figure 3C).

Cytotoxicity was further evaluated by live/dead staining. In the PEEK group, abundant PI-positive cells were observed, indicating higher cytotoxicity. The SPEEK group exhibited fewer dead cells (*p* < 0.05). In contrast, the SPEEK-GO-PEG and SPEEK-(GO-PEG/pBMP-2) groups showed negligible PI staining, demonstrating markedly reduced cytotoxicity (*p* < 0.001) (Figures 3D,E).

### 3.5 Osteogenic potential of SPEEK-(GO-PEG/pBMP-2)

**Figure 4:**
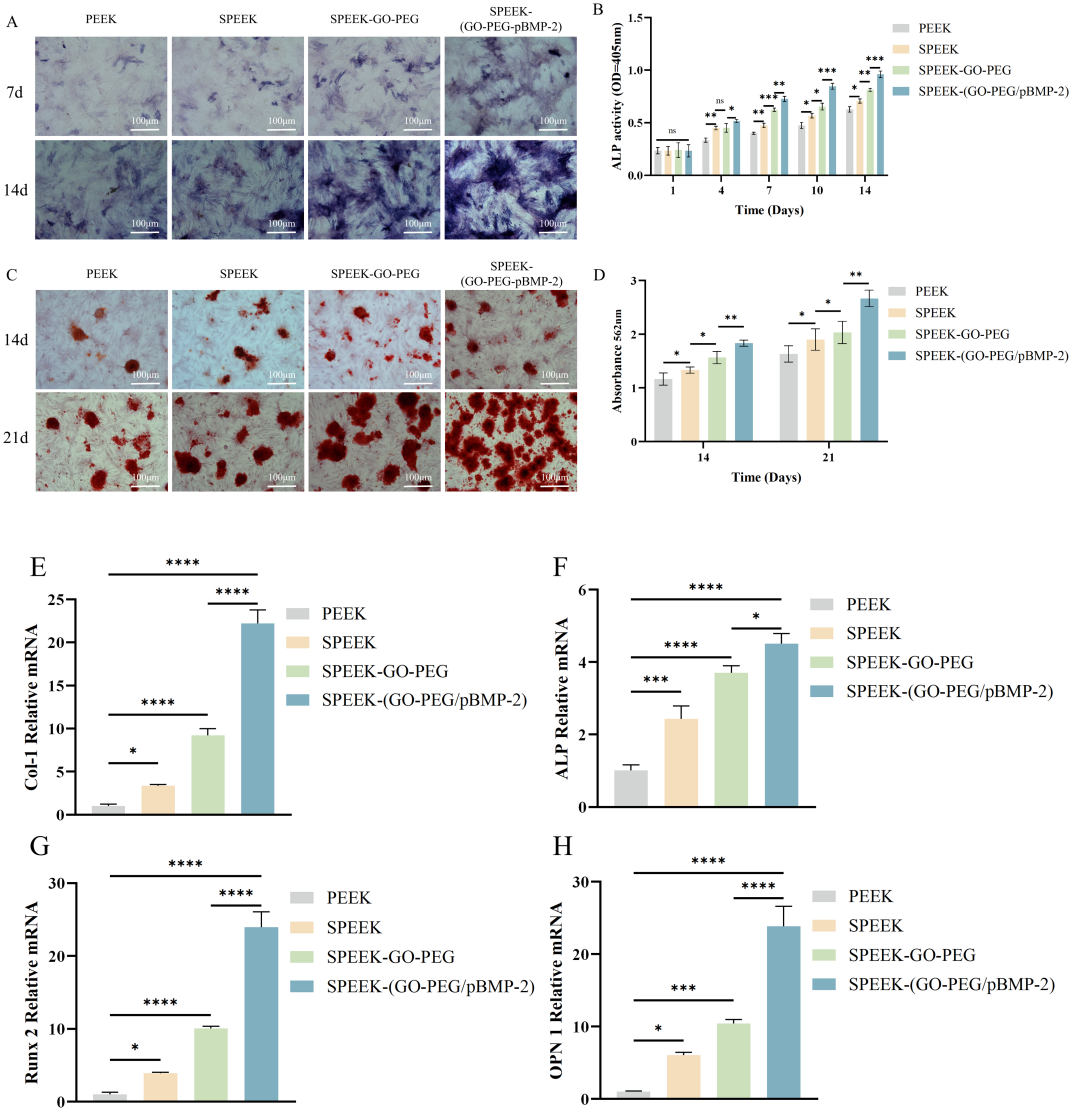
Osteogenic properties of SPEEK-(GO-PEG/pBMP-2). **(A)** ALP staining of rBMSCs cultured on different substrates. **(B)** Quantitative analysis of ALP activity. **(C)** Alizarin Red S staining of mineralized nodules. **(D)** Semi-quantitative analysis of mineral deposition. **(E–H)** qRT-PCR analysis of osteogenesis-related genes: **(E)** COL-1, **(F)** ALP, **(G)** Runx2, and **(H)** OPN. (ns, *p* > 0.05; *p < 0.05; **p < 0.01; ***p < 0.001; ****p < 0.0001).

After 7 and 14 days of osteogenic induction, ALP staining revealed blue–purple signals in all groups, with intensity increasing over time. At both time points, the SPEEK-(GO-PEG/pBMP-2) group displayed markedly stronger staining than the other groups, indicating the highest ALP expression (Figure 4A). Consistently, ALP activity assays performed at days 1, 4, 7, 10, and 14 showed no significant differences among groups on day 1. From day 4 onward, ALP activity in the SPEEK-(GO-PEG/pBMP-2) group was significantly higher than in all other groups (*p* < 0.001), confirming superior osteogenic activity (Figure 4B).

After 14 and 21 days of osteogenic induction, alizarin red staining revealed orange–red mineralized nodules in all groups, with staining intensity increasing over time. At both time points, the SPEEK-(GO-PEG/pBMP-2) group exhibited markedly stronger staining and a greater number of mineralized nodules than the other groups. Semi-quantitative analysis confirmed these findings, showing significantly higher mineral deposition in the SPEEK-(GO-PEG/pBMP-2) group (*p* < 0.05) (Figures 4C,D).

The osteoinductive effects of the composites were further assessed by examining the expression of osteogenesis-related genes, including COL-1, ALP, Runx2, and OPN, using qRT-PCR, with expression levels normalized to GAPDH. Gene expression profiles across the four material groups were consistent with the osteogenic staining results. Notably, the SPEEK-(GO-PEG/pBMP-2) group exhibited significantly higher expression of COL-1, ALP, Runx2, and OPN compared with the other groups (*p* < 0.05) (Figures 4E–H).

### 3.6 Antibacterial activity of SPEEK-(GO-PEG/pBMP-2)

**Figure 5:**
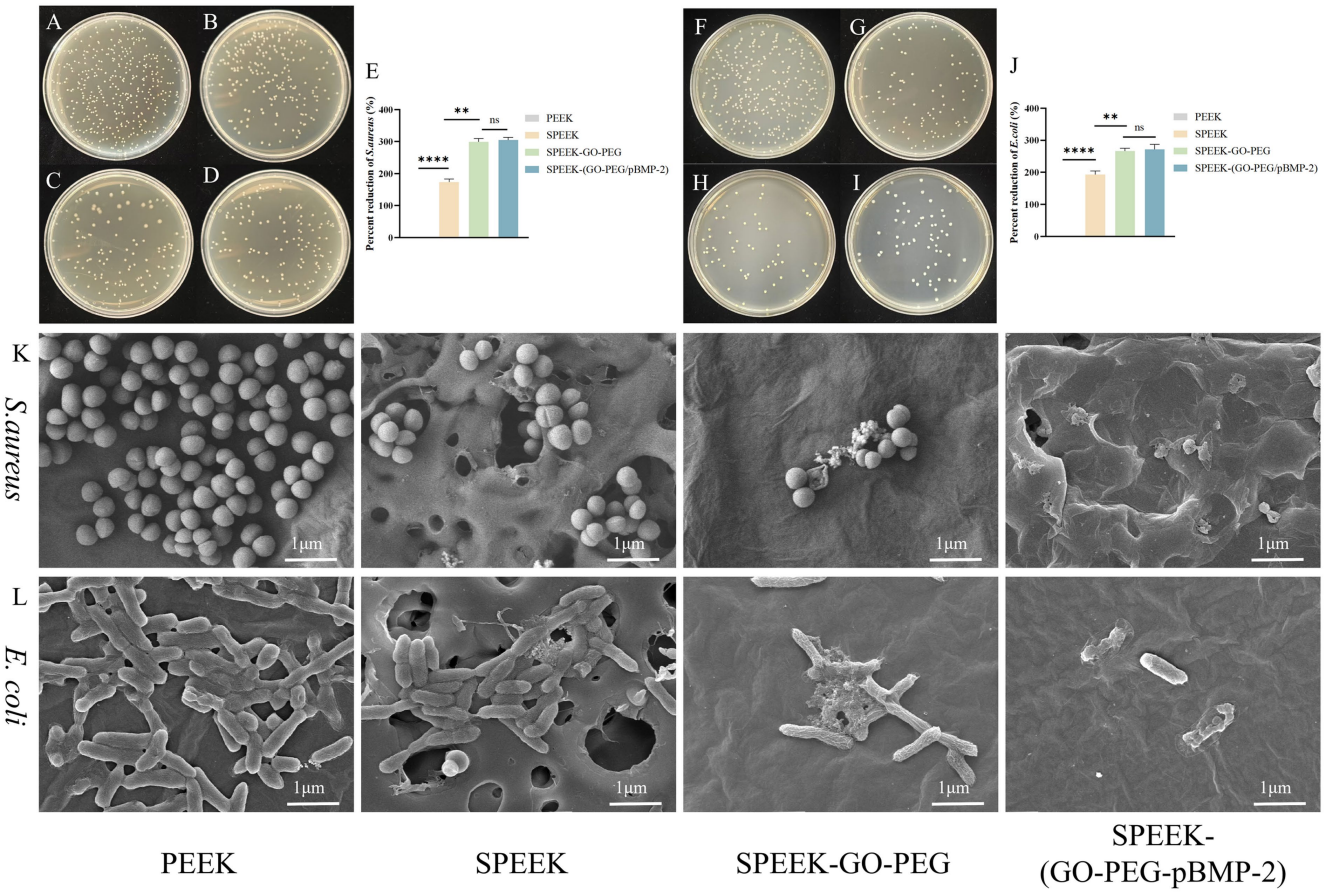
Antibacterial activity of SPEEK-(GO-PEG/pBMP-2). **(A–D)** Colony counts of *S. aureus* cultured on PEEK, SPEEK, SPEEK-GO-PEG, and SPEEK-(GO-PEG/pBMP-2). **(E)** Quantitative analysis of *S. aureus* colony counts. **(F–I)** Colony counts of *E. coli* cultured on different substrates. **(J)** Quantitative analysis of *E. coli* colony counts. **(K)** Representative SEM images showing *S. aureus* morphology on material surfaces. **(L)** Representative SEM images showing *E. coli* morphology on material surfaces. (ns, *p* > 0.05; **p < 0.01; ****p < 0.0001).

The antibacterial properties of the materials against *S. aureus* and *E. coli* were assessed using the plate count method. Compared with PEEK, all modified groups exhibited enhanced antibacterial activity. Both SPEEK-GO-PEG and SPEEK-(GO-PEG/pBMP-2) demonstrated stronger antibacterial effects than SPEEK, with no significant difference between the two groups (*p* < 0.01) (Figures 5A–J).

SEM was used to further evaluate bacterial morphology on the surfaces of different materials. For *S. aureus*, bacteria on PEEK exhibited intact spherical morphology, with many in the division phase and densely distributed across the surface, indicating active proliferation. On SPEEK, bacterial numbers decreased, but morphology remained largely unchanged, and distribution was irregular due to the porous structure. In contrast, SPEEK-GO-PEG and SPEEK-(GO-PEG/pBMP-2) surfaces showed markedly reduced bacterial counts. Most bacteria displayed morphological alterations, including wrinkled envelopes, with partial membrane rupture observed in some cells. For *E. coli*, bacteria on PEEK exhibited intact rod-shaped morphology with smooth edges and dense surface coverage. On SPEEK, bacterial numbers decreased without major morphological changes. On SPEEK-GO-PEG and SPEEK-(GO-PEG/pBMP-2), bacteria exhibited visible membrane indentations, irregular edges, and surface wrinkling (Figures 5K,L).

## 4 Discussion

Despite substantial advances in dental and orthopedic implantology, long-term implant success remains limited by two mechanistically intertwined challenges: insufficient osseointegration and implant-associated infections (IAIs) (4). Suboptimal early interfacial events—including unfavorable protein adsorption, impaired cell adhesion, and dysregulated inflammatory–osteogenic balance—can compromise bone formation and mechanical stability (24). When interfacial bone regeneration fails to withstand functional loading, micromotion and fibrous encapsulation ensue, ultimately jeopardizing secondary stability (25). IAIs originate from bacterial adhesion to the conditioning film on implant surfaces, followed by biofilm maturation that confers resistance to immune clearance and antibiotic therapy (26). The resulting chronic inflammatory milieu suppresses osteoblast activity while enhancing osteoclastogenesis and peri-implant bone resorption. Importantly, these processes are mutually reinforcing: compromised osseointegration facilitates bacterial colonization, whereas infection-driven inflammation further impairs bone remodeling, establishing a pathological feedback loop that predisposes implants to failure (27).

A variety of implant materials—including titanium alloys, hydroxyapatite ceramics, and biodegradable polymers—have been developed to address mechanical and biological demands (28). Metallic systems provide mechanical robustness but suffer from radiopacity and stress shielding; bioactive ceramics offer osteoconductivity yet lack fracture resistance; biodegradable polymers enable temporary support but are insufficient for long-term load-bearing applications (29, 30). Among polymeric materials, PEEK has gained considerable attention owing to its bone-matched elastic modulus, radiolucency, and chemical stability. Nevertheless, its biological inertness and hydrophobicity limit protein-mediated cell adhesion and osteogenic signaling, while the absence of intrinsic antibacterial activity renders it vulnerable to bacterial colonization (1, 7).

To overcome these limitations, diverse PEEK surface modification strategies have been proposed, including metallic deposition, inorganic coatings, carbon-based nanomaterials, polymer grafting, and growth factor immobilization (31). Among them, bioactive coatings with defined composition and hierarchical architecture are particularly effective in modulating cell–material interactions at the molecular scale (32). However, direct delivery of recombinant growth factors is frequently constrained by rapid degradation, short biological half-life, and burst release (20). Consequently, carrier systems capable of stabilizing bioactive molecules and enabling sustained release are of significant translational relevance.

Here, we employed PEG-functionalized graphene oxide (GO-PEG) as a gene delivery platform for pBMP-2 and integrated it onto a sulfonated PEEK (SPEEK) substrate. PEG conjugation enhanced GO dispersibility and cytocompatibility while preserving reactive functional groups for efficient plasmid loading. An optimized N/P ratio of 20 achieved a balance between transfection efficiency and cell viability, suggesting that excessive carrier content may induce cytotoxic stress. Upon immobilization onto SPEEK, the GO-PEG/pBMP-2 coating significantly improved cellular adhesion and spreading. SEM and cytoskeletal imaging revealed well-organized F-actin structures and enhanced cell–substrate interactions, indicative of stable focal adhesion formation and favorable mechanotransduction signaling.

Importantly, the composite exhibited a sustained release profile of pBMP-2 *in vitro*. The gene loading efficiency reached 1.4% ± 0.5%, and the release behavior showed an initial burst phase followed by prolonged and controlled release, with cumulative release reaching approximately 79% by day 30. Such sustained plasmid availability at the cell–implant interface may support continuous transfection of rBMSCs and subsequent endogenous production of therapeutic BMP-2 protein. BMP-2 is known to enhance ALP expression and calcium phosphate deposition by activating multiple osteogenic signaling pathways, including BMP-2/Smads/Runx2/Osterix, Wnt/*β*-catenin, p38 MAPK, and ERK1/2 (33).

Consistent with this prolonged osteogenic stimulation, early upregulation of Runx2, COL-1, ALP, and OPN was observed at day 7, indicating initiation of osteogenic commitment. Elevated ALP activity at days 7 and 14 further confirmed sustained differentiation. Robust mineralized matrix deposition at days 14 and 21 suggested maintained osteoinductive signaling during late-stage maturation. Collectively, the integration of controlled gene delivery with a structurally bioactive surface establishes a synergistic microenvironment conducive to bone regeneration.

Beyond osteogenesis, GO incorporation conferred pronounced antibacterial activity against both *S. aureus* and *E. coli*. Reduced colony formation and SEM-detected membrane disruption support established mechanisms of GO-mediated antibacterial action, including physical membrane damage, oxidative stress induction, and electron transfer interference (14). Notably, incorporation of pBMP-2 did not compromise antibacterial efficacy, indicating that antibacterial activity was primarily attributable to the GO component.

Compared with conventional surface modification strategies—such as metallic coatings or passive adsorption systems—the present multifunctional platform integrates osteogenic stimulation and antibacterial protection within a unified architecture. Clinically, such dual functionality may reduce early implant failure by simultaneously promoting stable osseointegration and limiting microbial colonization. By decreasing dependence on systemic antibiotics or repeated interventions, this strategy may ultimately enhance implant longevity and reduce complication rates in both dental and orthopedic settings.

Although the present study demonstrates promising *in vitro* performance, translation to *in vivo* applications presents additional challenges. The physiological environment—characterized by blood flow, immune responses, protein adsorption, and mechanical loading—may influence coating stability, degradation behavior, and gene transfection efficiency (34). Immune cell recruitment and protein corona formation could potentially alter release kinetics and reduce local gene availability (35), while mechanical stresses during surgical insertion and at the bone–implant interface may affect coating integrity and long-term durability (36).

In the present study, preserved surface morphology following in vitro culture, together with the sustained 30-day release profile, suggests structural and functional stability of the coating under aqueous conditions. The porous SPEEK substrate, combined with the freeze-drying immobilization strategy, may facilitate mechanical interlocking and interfacial interactions that contribute to coating retention. Nevertheless, quantitative adhesion testing under simulated shear stress was not performed in the present study and warrants further investigation.

To comprehensively evaluate translational potential, future studies will incorporate mechanical adhesion assessments, including scratch resistance and dynamic wash-off assays, alongside established animal implantation models. Osseointegration will be analyzed using micro-CT, histological staining, and mechanical push-out testing, while infection models will be introduced to verify antibacterial efficacy *in vivo*. These investigations will provide critical insights into coating stability, gene delivery efficiency, and biological safety within a dynamic physiological environment.

## Data Availability

The original contributions presented in the study are included in the article/Supplementary material, further inquiries can be directed to the corresponding author.

## Supplementary material

Supplementary Figure S1 The BMP-2 release profile.
