# Supplementary figures and images for: Dual-functional sulfonated PEEK implants via graphene oxide–mediated BMP-2 gene delivery: enhanced osteogenic and antibacterial performance in vitro
Source: Front Med (Lausanne). 2026 Mar 4;13:1763692. doi: 10.3389/fmed.2026.1763692 (PMC12996093; doi:10.3389/fmed.2026.1763692)

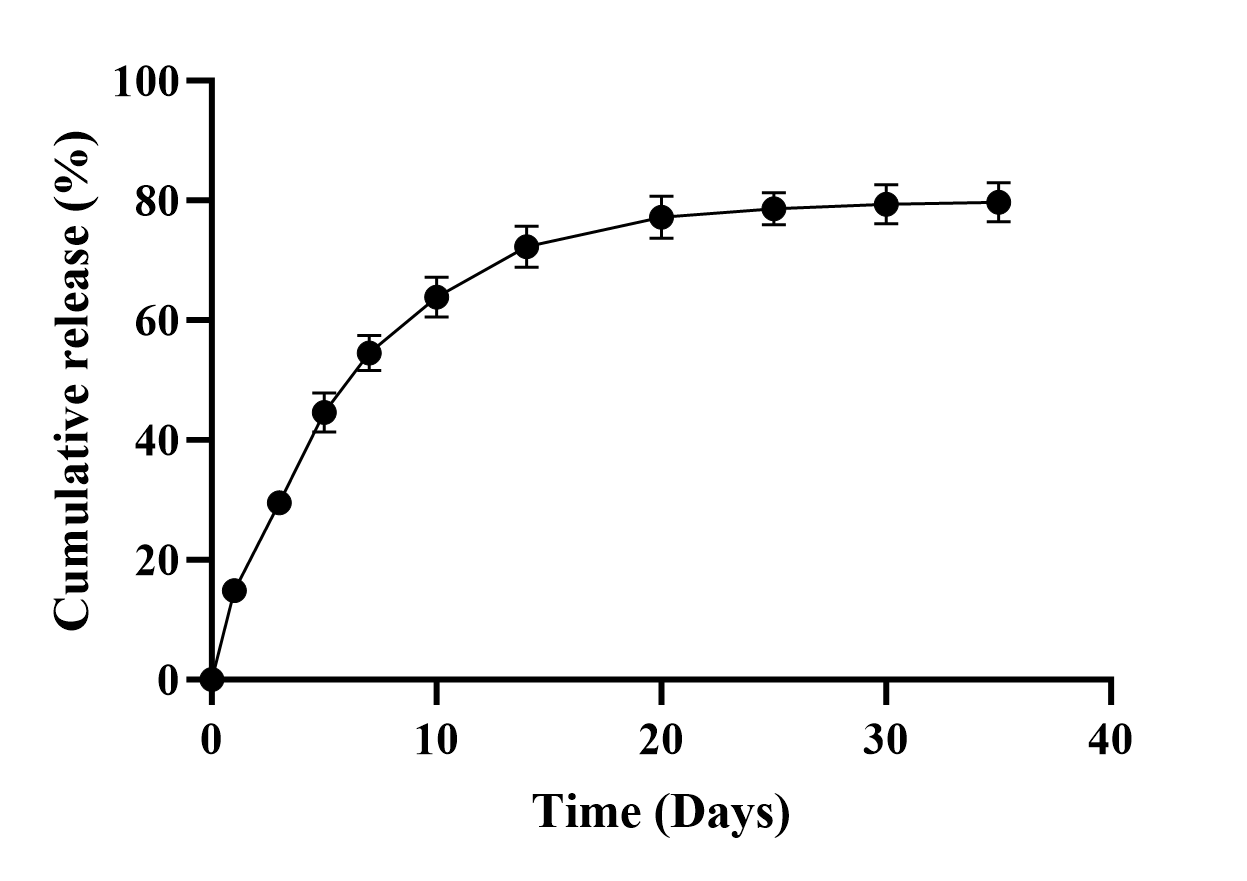

Supplement: Supplementary Figure S1 — The BMP-2 release profile. [file Image_1.tif]
